# Towards Social Radiology as an Information Infrastructure: Reconciling the Local With the Global

**DOI:** 10.2196/medinform.3648

**Published:** 2014-10-03

**Authors:** Gustavo Henrique Matos Bezerra Motta

**Affiliations:** ^1^Centro de InformáticaDepartamento de InformáticaUniversidade Federal da ParaíbaJoão PessoaBrazil

**Keywords:** information infrastructures, social radiology, teleradiology, picture archive and communication systems (PACS), sociotechnical systems

## Abstract

The current widespread use of medical images and imaging procedures in clinical practice and patient diagnosis has brought about an increase in the demand for sharing medical imaging studies among health professionals in an easy and effective manner. This article reveals the existence of a polarization between the local and global demands for radiology practice. While there are no major barriers for sharing such studies, when access is made from a (local) picture archive and communication system (PACS) within the domain of a healthcare organization, there are a number of impediments for sharing studies among health professionals on a global scale. Social radiology as an information infrastructure involves the notion of a shared infrastructure as a public good, affording a social space where people, organizations and technical components may spontaneously form associations in order to share clinical information linked to patient care and radiology practice. This article shows however, that such polarization establishes a tension between local and global demands, which hinders the emergence of social radiology as an information infrastructure. Based on an analysis of the social space for radiology practice, the present article has observed that this tension persists due to the inertia of a locally installed base in radiology departments, for which common teleradiology models are not truly capable of reorganizing as a global social space for radiology practice. Reconciling the local with the global signifies integrating PACS and teleradiology into an evolving, secure, heterogeneous, shared, open information infrastructure where the conceptual boundaries between (local) PACS and (global) teleradiology are transparent, signaling the emergence of social radiology as an information infrastructure.

##  Introduction

Contemplating social radiology in terms of an information infrastructure [1-7] goes beyond discussion on technologies for archiving and transmitting medical images or tools for medical imaging. It involves the notion of a shared infrastructure as a public good [2], capable of supporting the formation of associations between people, organizations and technical components in order to support patient care and radiology practice in a networked world. The emergence and sustainability of an information infrastructure require a permanent endeavor in order to build and maintain a set of solutions distributed along the social or technical and local or global axes of the information infrastructure space [2]. When solutions polarize, emphasizing the local (technical) aspect rather than the global (social), or vice-versa, the information infrastructure does not emerge. In a recent work [5], the term “knowledge infrastructure” is used as new terminology for “information infrastructure”.

This article reveals the existence of a polarization between the local and global demands for radiology practice, thus jeopardizing the emergence of social radiology as an information infrastructure. The article begins by demonstrating that such polarization establishes a tension between local and global radiology practices and that this tension denotes the lack of an information infrastructure. The following explores the concept of an information infrastructure for radiology practice as a social space that is interactive, evolving, and open. Next, it analyses how the struggle with the inertia of the installed base impedes the emergence of social radiology as an information infrastructure. Additionally, it also argues that current teleradiology models do not configure as an information infrastructure for social radiology. The article concludes by discussing the manner in which reconciliation between local and global is a way towards social radiology as an information infrastructure.

##  The Tension Between Local and Global Radiology Practices

Sharing medical imaging studies among health professionals in an easy and effective manner has long been an on-going pursuit in radiology [8]. This is even more evident nowadays, with the widespread use of medical images and imaging procedures in clinical practice and patient diagnosis. The demand for noninvasive diagnostic imaging tests continues to increase, where the growing trend among non-radiologist physicians is twice as fast as among radiologists [9]. As such, the timely access to medical imaging studies by radiologists and non-radiologists is imperative.

In general, radiologists have no major issues with reading images and creating primary diagnostic reports. Similarly, other health professionals have no concerns about reading such images and reports when access is within the domain of a health care organization. Image related data and working functions are accessed in a workflow supported by the picture archive and communication systems (PACS) and radiology information systems (RIS) of a radiology department [10,11]. When access is required from a remote location, health care organizations typically adopt a suitable teleradiology solution. For instance, they adopt virtual private networks (VPN) and cloud computing technologies to enable physicians to access PACS/RIS from a different location or to integrate geographically separated buildings within a health care organization [12-14]. Another solution commonly used is outsourcing image interpretation services. In this case, regional, national or international teleradiology companies only interpret or broker image interpretation for non-radiologists [15].

The big issues occur when health professionals need to access medical image studies outside the domain of a health care organization. Specifically, it is not easy for a physician to share an image study effectively for a second opinion with a colleague situated in a different location. By contrast, when all the actors are in the same (local) domain, it is easier to share the study through the radiology workflow of PACS/RIS. It is also easy to distribute finished reports to the referring physicians once they are in the same domain. In spite of advances in federated teleradiology solutions for integrating image sharing among health care organizations [16], they are complex to implement and such alliances involve business models. Ultimately, it would be of little interest for competing teleradiology companies to share medical imaging studies and other collaborative resources among themselves. On the other hand, the care of the patient should be of paramount interest for sharing medical imaging studies, so as to have an expert second opinion on a complex case. In such a situation, the consultant physician generally chooses the expert radiologist in a rather arbitrary manner, regardless of any kind of business alliances that may exist among health care organizations. Selection of the expert radiologist, as an illustration, may be based on the consultant physician’s professional relationships, or on the recommendation of colleagues, or through reputation within a subspecialty. Essentially, there are many factors that affect the sharing of imaging studies for patient care, which require the flexibility and dynamism of teleradiology infrastructure to support the spontaneous formation of temporary or permanent practice alliances of health professionals and organizations. However, global health initiatives often adopt highly centralized or rigidly hierarchical approaches for scaling up health services, which are not fitting for the dynamic, unpredictable manners in which health services may expand and become sustainable [17]. In particular, teleradiology infrastructures of health care organizations tend to be tailored to satisfy local requirements. For instance, providing image interpretation or second opinion advice services to previously defined remote locations as part of a locally managed teleradiology service or, on the other hand, acting as a user of services provided by a specific teleradiology company. This leads to an emphasis on detailed initial planning and inflexible designs, which do not address the adaptive properties of dynamic pathways for expanding health services [17].

Despite the weaknesses of using email, in teleradiology it has become the most popular way to overcome this lack of flexibility and dynamism [18]. In its simplest form, the physician just collects the images of interest into some well-known format (eg, JPEG), packs and emails them to a remote expert, who will then review the images and reply with a report. In a more advanced manner, the registered DICOM MIME type [19] allows the transfer of imaging studies in DICOM standard format [20] using basic email transport mechanisms with additional encryption in accordance with OpenPGP [21]. Weisser et al [22] present the successful experience of integrating more than 60 health care organizations in Germany by transmitting DICOM imaging studies via email in a variety of teleradiology applications. Email has also been successfully used to send reports to women undergoing mammography screening in the United States [23]. Hence, what does the use of email in radiology suggest? It suggests email is a simple way to connect people and exchange medical imaging studies beyond the limited boundaries of local PACS networks. With email, it is easy to locate and connect people in order to exchange images or reports and collaborate asynchronously thanks to the simplicity, availability, connectivity, large number of users and low cost of email. Pianykh [18] claims that email radiology was “the first honest attempt to implement true teleradiology”; however, he also recognizes the drawbacks such as, poor image quality, the loss of metadata when images are converted from DICOM to common image formats (eg, JPEG), difficulties in dealing with large files, and a lack of any PACS/RIS integration and consolidated workflow.

**Figure 1:**
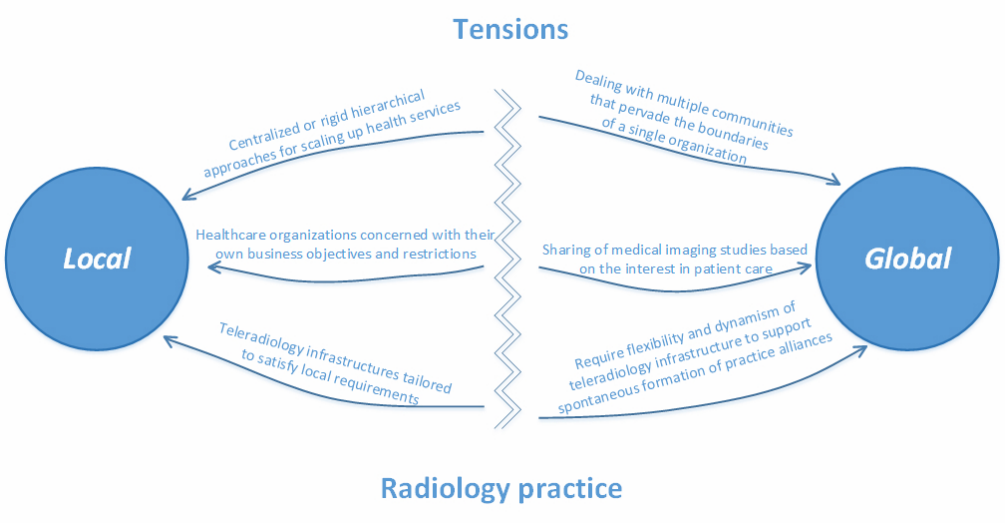
Polarization due to tensions between local and global demands for radiology practice.

It is manifest that there is a polarization between the local and global practice needs (Figure 1). In general, the local needs of the radiology practice are well afforded by the radiology workflow of the PACS/RIS in a health care organization, whereas there is a great impediment to support the global needs. This impediment is motivated by a radical difference between the local and global needs of the radiology practice. Health care organizations are often concerned with their own business objectives and restrictions, which influence the working practice of the local radiology community as well as the local PACS/RIS infrastructure. On the other hand, individuals, radiologists, patients and the many other stakeholders in the health care system are often members of multiple communities that pervade the boundaries of a single organization, interacting with one another through a web of complicated relationships influenced by communities of practice, neighborhoods and social networks [17]. Moreover, each practice community uses technologies differently, thus presenting different demands on their flexible standard requirements [7].

Such polarization establishes a tension between the local and global demands (ie, the demands that encompass the boundaries of a single organization) that denotes a lack of an information infrastructure for the radiology practice and this very infrastructure will occur only when this tension is resolved [2,7] by reconciling the local with the global. However, the emergence of such an information infrastructure is a long-term venture, which requires that it is considered not as something entirely transparent and ready to run or operate as something else, but as a social space of interrelations between people, organizations and technical components [2]. In fact, information infrastructures emerge not by emphasizing changes in the infrastructural components but from changes in the infrastructural relations, since information infrastructures are fundamentally a relational concept [2,7]. In this sense, to be social does not signify being a thing among other things, but a kind of association between things that are not themselves social, a movement that may fail to trace any new association and may fail to redesign any well-formed assemblage [24].

## Information Infrastructure for Radiology Practice as a Social Space

### Overview

The information infrastructure for the practice of radiology means a social space of static and dynamic interactions where people, organizations and technical components are associated with activities and structures, forming a sociotechnical system. This social space may be a physical place, such as a radiologist’s report room or a virtual space such as the radiology department, and it simultaneously offers material and immaterial support for social relations [25]. The material support for the practice of radiology, among others, includes physical rooms, furniture, medical imaging equipment, information technology (IT) devices, and networks. The immaterial support comprises business and clinical processes (activities) of the radiology department, organizational structure, roles and functions, IT and communication software (eg, PACS, RIS), among others. It is in this social space that people gather and interact with each other, with material and immaterial support. In addition, the social space for radiology practice is evolving and open.

### The Social Space for Radiology Practice is Interactive

The interactions that occur in the social space for radiology practice can vary from rather static to highly dynamic. For instance, the relationship between a radiologist and an image modality tends to be rather static. Specifically, an expert radiologist in nuclear medicine (NM) tends to have a static relationship with the NM division of the radiology department in the sense that they belong to this division in the organizational structure for an indefinite time. In fact, such experts tend to work with the same set of imaging equipment located in specific rooms of the NM division, use a familiar set of imaging manipulation tools and other information and communication technology (ICT) tools, carry out routine sets of clinical and administrative activities, and finally, usually cooperate daily with a well-known staff. In short, a health professional working in their practice in a social space establishes static relationships with material and immaterial support. In general, such relationships have a high inertia, change slowly over time, and are tied to a local context.

Other interactions are much more dynamic, such as the relationship between patients and the radiology department. Some patients go to the radiology department only once, while others make several visits during a short period of time, according to the condition of their health. Some visits are motivated by emergencies or the need for urgent examinations, while others are motivated by chronic diseases. One single patient may undergo tests with different imaging modalities, where images are acquired with the support of different kinds of equipment, clinical and administrative processes and various personnel, with the results being evaluated by different radiologists. Complex cases may require special care for patient preparation before the test, application of elaborate post-processing techniques on the acquired images, or the formation of medical boards to discuss findings. Certain demands, especially in emergencies or urgent examinations, may be highly dynamic and unpredictable. In sum, a patient in the social space of a radiology department establishes dynamic relationships with the physical environment, imaging modalities, software tools, clinical and administrative processes and personnel, among others. In general, the occurrence of such relationships is ephemeral and dependent on the patient’s health and financial condition, but the outcomes may have a significant repercussion on the patient’s life.

### The Social Space for Radiology Practice is Evolving

With the aim of considering the social space as something that is evolving, it is essential to highlight the relational role played by the interactions between people, organizations, and technical components. This shifts the emphasis away from things and people as simply being causal factors during the performance of such practices [7], since interactions generate a chain of actions and reactions along complex pathways that influence the evolution of the social space in a variety of manners. For instance, the radiology department (a virtual place) interacts with the physical building of the hospital into which it is located. In this case, the physical building is continually adapting to satisfy the requirements of the radiology department while on the other hand, the physical restrictions of the building continuously affect the work practices in an iterative and interwoven mode. Similarly, the technical components are affected by the requirements of the radiology department, as well as by interactions with people and things, such as other technical components. However, this process is not only one-way, and people also place their interests in the technology [26]. As an illustration, it is possible for technology to change common activities within the radiological workflow in order to adapt to its interests, where radiologists, for example, have to learn a new method of entering diagnoses and other information onto a structured reporting system that has replaced the traditional use of free text editors. Conversely, technical components are modified and adapted to accommodate the demands of people and things, such as complying with a technical standard (eg, DICOM, Health Level Seven [27]) or changing the procedures for patient appointments, allowing them to also be booked via a mobile phone app. In short, the conventions of practice both shape and are shaped by information infrastructure [7]. Indeed, from the interplay of people, organizations and technical components in the social space there emerges a concurrent design and redesign of technology, individuals and work practices [26], forming an ever-evolving sociotechnical system.

### The Social Space for Radiology Practice is Open

It is important to note that such interactions occur not only inside the local social space of a radiology department, but also within the external environment, since social spaces are inherently open, stretching outside their own location. This signifies that in spite of there being a boundary, a local social space is permeable to its external environment for the exchange of matter, energy and information. While on the one hand, such an exchange is essential for the social space of a radiology department to keep its internal organization and evolve, on the other, it also allows it to influence the (external) environment. However, being open does not denote being completely free because members of a social space have to adhere to rules, business and clinical processes, policies and principles, some self-determined and others determined from outside. For instance, not everyone is free to enter a radiology department to work as a radiologist. From an external perspective, there are legal requirements that the radiology department must observe regarding professional credentials in order to allow an applicant to work as a radiologist. However this is not enough. Based on its policies and principles, the radiology department itself should also have a particular interest in hiring a new radiologist, such as the need to expand the workforce to meet growing demands. Once hired, the radiologist becomes a member of a social space of work practice, in which membership signifies having to learn the rules [7], business and clinical processes, policies, and principles of the radiology department. Nonetheless, the radiologist, with their professional and personal history also has the potential to interfere and change the above mentioned items. Similarly, this also occurs with anyone who interacts with or within the social space of the radiology department, including other professionals and patients.

Openness can be helpful to deal with situations of emergency, particularly when they require a large effort in reading images that cannot be supported by a local radiology department alone. In this case, an open social space for radiology practice enables the temporary mobilization of a taskforce for reading images, with radiologists from the external environment being invited to join it. The transmission of DICOM image studies by email, like the experience related by Weisser et al [22], is an example of an open infrastructure for radiology practice that can help in such mobilization. In principle, any radiologist having an email account and a trustful digital certificate can join the effort, being able to receive studies to read and to send reports in a secure mode.

Other kinds of interactions with the external environment are more subtle, thanks to the openness of social spaces. Consider the case of clinical research carried out by a radiology department. The findings of such research need not be used only to improve the work practice locally, but also externally, since they are published through scientific conferences and journals and are kept in digital libraries alongside the findings of clinical research performed elsewhere, compounding a body of knowledge. Thus, local findings are potentially able to influence the work practices of other (local) radiology departments. However, it is worth noting that scientific societies, conferences, and journals mediate this flow of information between (local) radiology departments. In fact, they are also social spaces for radiology practice because people, organizations, and technical components interact when dealing with this body of knowledge. It is there that primary (eg, original papers) and secondary (eg, books, reviews, clinical guidelines, technical standards) research findings are discussed, peer-reviewed, and shared. As a social space, it is there that cooperation and competition takes place, recommendations are made, reputations are built, conflicts emerge, and consensus is reached. Furthermore, the body of knowledge collectively produced has an influence over the local work practices of radiology departments, where, in general, clinical research is conducted. This is feasible because the boundaries of social spaces are permeable to their external environment. Hence, at the same time, the environment is both intimate and foreign (it is part of a social space, yet it remains exterior to it), so that the intelligibility of a social space is encountered not only in the social space itself, but also in its relationship with the environment which is not simply of dependence: it is constitutive of it [28].

Essentially, the aforementioned social spaces, including their relationships with the environment, comprise an information infrastructure for radiology practice. In the past, the main form with which to share scientific information was through the means of physical delivery. Another way to share information was to attend scientific conferences or visit a radiology service. It may be stated that the sharing of scientific research information through the radiological social space was quite successful in the past. Nowadays, with advances in ICT, such sharing has largely improved, not only by the ease of access provided by digital networks and libraries, but also by the support provided by ICT in conducting scientific research itself. However, information published from clinical research has an important property: it is aggregated information. Usually, selected clinical and demographic data are collected from patients as part of clinical research protocol performed in a radiology department or within a group of radiology departments. Such information is usually processed by statistical methods, summarized and analyzed locally by those responsible for the research before the findings being published. Consequently, scientific papers present clinical information in a highly condensed and abstract manner. On one hand, this facilitates the spread of information throughout the radiological social space but on the other, it hinders access to private information from the patients who took part in the research. The outcome is that, taking into account the flow of scientific information in the radiological social space, there is no significant tension between local and global. The tension occurs when the clinical and demographic information that needs to be shared belongs to one identifiable individual.

## Social Radiology Information Infrastructure Wrestles With the Inertia of an Installed Base

According to Star and Ruhleder [7], an information infrastructure is built on an installed base and wrestles with the inertia of this base, inheriting both its strengths and shortcomings. In the case of scientific information flowing into the radiological social space, ICTs, particularly the Internet and the Web, were introduced within an installed base that was mostly transposed to a virtual world, such as e-mails, e-documents, digital libraries, e-subscriptions, e-publishers, e-readers, and information systems to support scientific workflow. In short, the ease with which scientific information was commonly exchanged among local social spaces became even greater after the advent of ICTs, due to its growing pervasiveness. The ICT infrastructure to support radiology practices that deal with personal and identifiable clinical and demographic information was also built on an installed base. However, in this case, the radiological workflow, which deals with this kind of information, was traditionally confined to the boundaries of the radiology department. Before the advent of ICTs, the steps of the radiological workflow were performed within the physical space of the radiology department, where the generated medical images were made available on film and reports were written on paper. For situations requiring the opinion of a remote subspecialist radiologist, as in complex cases, the transmission of medical images over distance (ie, teleradiology) was not common due to technical difficulties of transmission, high costs, and poor image quality. In general, medical boards were formed to discuss complex cases with radiologists and other physicians from the local practice community of the radiology department or hospital. Concerns with the violation of patient confidentiality due to leaking sensitive clinical information also contributed to keeping the imaging studies within the confines of the radiology department that produced them.

Therefore, when ICTs were introduced into the radiology department to deal with personal and identifiable clinical and demographic information, they were used to support radiology practices that usually worked on a local basis. The examples include: (1) medical images generated on film were replaced by direct digital capture to produce a digital image available in DICOM standard format; (2) management of the physical films of medical images in the radiology department was replaced by PACS working according to DICOM requirements for image communication between individual components, such as imaging equipment, diagnostic and post-processing workstations, archive systems, and image distribution workplaces [10,11]; (3) the radiological workflow was mostly transposed to RIS/PACS, comprising software modules for creating orders, scheduling, reading, reporting, medical coding, recording services, and interfacing for billing systems, among others [11].

For cases in which teleradiology was required, it was only in the early 2000s that ICTs were ready for real clinical applications [10]. In general, teleradiology activities were supported by projects that created advanced infrastructures, although they were not sustainable, since they depended on short-term external resources that did not remain available after the end of the project [17]. In addition, such activities were conducted outside the radiology department routine, and did not complement it or become integrated. Thrall [8] reminds us that certain teleradiology efforts from the 1960s until the mid-1990s presented a relatively low performance as the cost of computers and data transmission were high, image quality was poor, and logistics were cumbersome. These efforts were unsustainable without external funding, and the clinical applicability in radiology work practices was very limited. By contrast, since the mid-1990s, particularly after the early 2000s, the evolution of ICTs provided a set of enhancements that enabled, in principle, an effective, sustainable use of teleradiology [8] as exemplified: (1) the availability of high-performance/low-cost personal workstations for image processing and display; (2) the availability of high-performance/low-cost storage and communications/computer networks like the Internet; (3) improvements in image compression algorithms and transmission techniques; (4) widespread use of PACS/DICOM by radiology departments.

While there is a inertia that confines the radiology work practices to the local social space of the radiology department, even after the arrival of ICTs the aforementioned enhancements bring very attractive opportunities to displace such work practices from the local to the global. In other words, they offer opportunities to disrupt the physical contiguity of the place where radiology work practices are usually performed. Fragments of the place need to be rearranged into a network in order to allow continuity of the work practices. In fact, there is still a movement in progress towards changing from a space of places to a space of flows, in terms of Castells’ nomenclature [29]. The space of places (ie, the local social space of the radiology department) organizes experience and activity around the confines of a locality, while the space of flows electronically associates separate places in an interactive network that connects activities and people in distinct geographical contexts [30], that is, the global social space for radiology practice.

##  Current Teleradiology Models are not Information Infrastructures for Social Radiology

The movement against local inertia does not result in the social space vanishing from the radiology department. In fact, it results in its transformation by the new possibilities of organizing people, activities and structures. Today’s common teleradiology models illustrate some of these possibilities [8,10,18]:

1. *Night-hawking/On-call/Off-hour reading*: these terms refer to providing on-call coverage for image interpretation, particularly during off-hours, when the availability of radiologists on examination sites is scarce. It is clear in the model that the radiology department has its own staff of radiologists, and on-call coverage is provided by designated members of the staff or by outsourced radiologists from a teleradiology company. In this last case, it is common to read images overnight in another country, taking advantage of a different time zone and lower costs.
2. *Regional PACS*: this model uses WAN to integrate local PACS or DICOM workstations from remote locations. It is typical for inter-hospital PACS for instance, when hospitals or health care centers have branches or satellite image centers, when they form business alliances, or when they are under the umbrella of a large public health system. It is a current solution for developing national and international radiology networks.
3. *Radiology outsourcing*: a model in which a teleradiology company takes care of the radiology service when interpretations are not available on site. In general, the hired company is in a cost-efficient location and may provide teleradiology equipment, image storage and technical support in addition to remote image interpretation by radiologists.

Although these models impact the inertia that confines the radiology work practices to the radiology department, they do not truly configure as an emergent information infrastructure for social radiology.

For the above listed items, in model (1), teleradiology does not substantially affect the usual work practices of the radiology department that takes advantage of it in two ways [8]. Firstly, by offering timely radiology coverage to referring physicians and patients, regardless of the availability of internal on-site staff, and secondly, by improving the usage of the workforce when the radiology department has its own 24-hour staff coverage, taking advantage of this to offer image interpretation services to third parties. In short, teleradiology model (1) is used as a convenience, to enhance the usage of the local workforce, or to maintain a reasonable work life for local staff.

Teleradiology model (2) lacks the flexibility required by an information infrastructure. The integration of several local PACS is driven by the business concerns of a relatively small group of hospitals or health care centers, not by the true concern for sharing medical images in general. The difficulties of sharing images outside the regional PACS domain remain, as much of the conventional PACS inertia is inherited [18]. For instance, a common approach is to have a VPN via WAN connecting branches and satellite image centers to the central PACS of a main hospital. In this case, a regional PACS is essentially a conventional, but huge PACS [18]. For the case of business alliances involving few hospitals, customized solutions to integrate their PACS are common, but there are problems of interoperability. The alignment of business interests among the participants of a regional PACS, on the other hand, facilitates sharing efforts and cooperation because a trust relationship is a priori established. In spite of this, the regional PACS is merely an integration of local PACS among organizations with a great interest in such sharing, offering nothing new in relation to conventional PACS. High inertia to planning and maintaining regional PACS hampers the flexibility and dynamism required to support the spontaneous formation of temporary or permanent practice alliances of health professionals and organizations motivated by the interest in patient care. In sum, teleradiology model (2) does not significantly affect local work practices, nor does it facilitate the exploration of new possibilities for radiology work practices outside the domain of regional PACS members.

Finally, the main problem in model (3) is the dependence on a single company that, in general, provides an ad hoc infrastructure for teleradiology suited for making easy, fast, and cheap connections with clients. The design of such solutions tends to be inflexible, and does not address the complexities of interoperability, because teleradiology companies generally have no interest in sharing images with external entities. Even so, this model of teleradiology may impact the local social space of radiology work practice because it displaces the radiologists from the point of patient care to another location. Motivations for this displacement (ie, the absence of interpretations on site) are of a logistical and economic nature. Logistical motivation is a classic case for employing teleradiology: remote areas (eg, rural, difficult to reach and possibly with only non-radiologist physicians) using a remote service for image interpretation by radiologists, including emergency situations and for second opinions. The impact in this case is positive. The economic motivation, on the other hand, aims to reduce the costs of maintaining an onsite team of radiologists. Local staff is replaced by remote radiologists hired by teleradiology companies situated in a cost-efficient location. One criticism of this last motivation is that it leads radiology work practice towards commoditization (assembly-line approach), as teleradiology companies and hospitals seek to maximize financial gain, without due concern for consultative skills, the necessary assessment or quality control provided by radiologists [15].

## Reconciling the Global With the Local

Essentially, in such common teleradiology models, work practice from the confines of locality is not truly reconciled into a space of flows to form a global social space for radiology. While they fragment the physical contiguity of the place where radiology work practice is performed, such fragments somehow remain close, due to local inertia. As a result, the impediment of sharing medical imaging studies with other localities remains high, since tension between the local and global persists, thus hindering the emergence of social radiology as an information infrastructure.

Observing from the perspective of Marc Berg’s Rationalizing Medicine [31], there was a convergence between technological tools and radiology practice when ICTs were introduced in radiology departments. The very creation of the DICOM standard, PACS and RIS as well as new or reshaped radiology practices, commonly found today on local radiology departments, was “not pre-given, but emerged in and through the development and intertwining of networks” [31], involving health professionals, technicians, patients, organizations, among other stakeholders. Such convergence, on the other hand, was not observed in the common teleradiology models presented beforehand in order to signal a seamless integration of local and global into an emerging information infrastructure for radiology practice. This suggests that such models are some of the “many loose ends” that confronts processes of convergence [31], still in progress towards social radiology information infrastructure.

An effective information infrastructure for radiology practice should facilitate social interaction regardless of any kind of business alliances among health care organizations. Here, reconciliation between local and global signifies teleradiology as an integral part of PACS, being the notion of (local) PACS and (global) teleradiology transparent, with digital imaging without the constraints of distance becoming a true radiology standard [18]. In fact, this facilitation will be reached insofar as the information infrastructure is shared, open, heterogeneous, secure, and evolving, forming a sociotechnical system of information technology.

A shared information infrastructure means considering it as a public good [2], and not belonging to a single company or organization, but shared across multiple communities in many, unexpected ways [1]. An open information infrastructure means that it has permeable boundaries, which allow interactions with an external environment in intricate, unexpected manners and contexts. In fact, the boundaries are not clear enough to distinguish those that may use the information infrastructure and those that may not, nor those that may design the information infrastructure and those that may not [1]. Heterogeneity reflects the great social and technical diversity afforded by an open, shared information infrastructure, able to include a growing number of entities such as user communities, operators, governance and standardization bodies, and design communities [1,4]. A secure information infrastructure signifies the capability of establishing trust among the entities of which it is composed, in consideration of legal and ethical issues such as patient privacy, confidentiality, integrity and ownership of clinical data, licensure, accreditation and liability of health professionals and organizations [16]. The experience of the DICOM email in Germany [22] is an example of an open and loosely coupled infrastructure for teleradiology that addressed such security issues. Finally, an evolving information infrastructure signifies considering it as emerging from the continuous interplay of people, organizations and technical components in a concurrent process of design and redesign [26].

**Figure 2:**
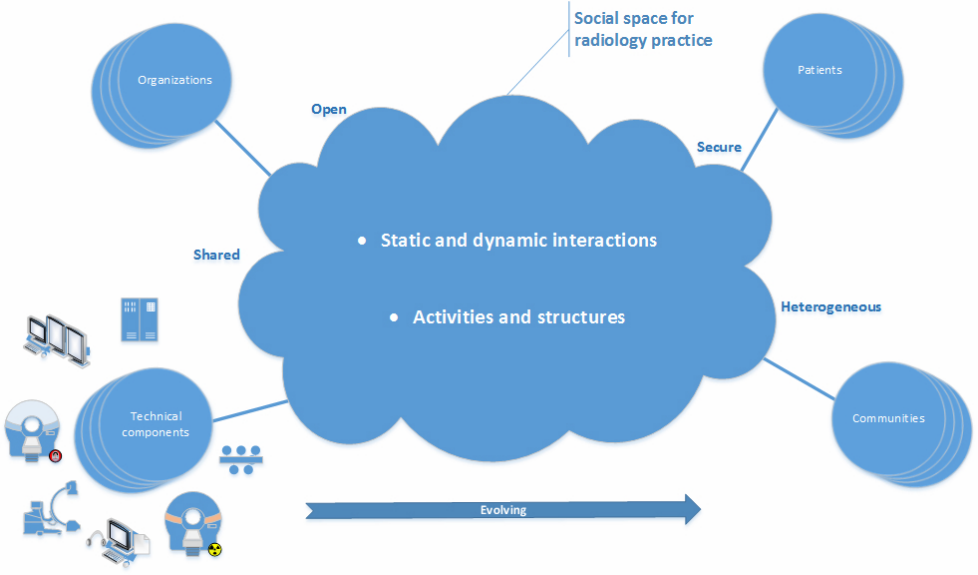
Social radiology as an information infrastructure.

In point of fact, social radiology as an information infrastructure (Figure 2) is a social space of static and dynamic interactions for radiology practice where people, organizations and technical components are associated to activities and structures forming a sociotechnical system of information technology that is shared, open, heterogeneous, secure, and evolving. It may enable the reorganization of radiology work practice into cyberspace (space of flows) to form a global social space for radiology that surpasses current teleradiology models. As a sociotechnical system of information technology, it is a basis for social computing that may provide value way beyond that offered by purely IT systems, since user-generated content is exploitable not only by the users, but by the information infrastructure itself [32].

The information infrastructure may be of value by producing faster results due to multiplying effort [32]. For instance, the information infrastructure may facilitate the spontaneous formation of small groups of subspecialist radiologists to provide expert consultations [15]. The agglutination of such groups to form larger groups may additionally provide 24/7 coverage for several small organizations and thus, the responsibility for off-hour emergency examinations, shared and spread over a large number of people, is able to enhance productivity of local workforce usage while maintaining a reasonable work load [33]. By being open and shared, a social radiology information infrastructure empowers radiologists to come together to provide professional services without the need of a teleradiology company acting as a broker.

The information infrastructure may also be of value by producing high quality results because it enables the integration of knowledge from multiple professionals with diverse expertise [32]. The existence of networks of groups of subspecialists favors the development of a culture of reciprocity in asking colleagues for advice and second opinions [33]. Indeed, it favors the creation of new models for assessing the quality of the radiologist’s work and for peer review [34], as they have been challenged by referring physicians and health care organizations to demonstrate the quality and accuracy of their interpretations more objectively [8]. This may result in solutions that tackle the problem of commoditization in radiology by enhancing the work of the radiologists while considering patient benefit essential [15]. In addition, the pursuing for quality favors groups of subspecialists to create their own culture and standards for reading images. As such, the different cultures for reading images present in local radiology departments can also happen in the cyberspace because an open, heterogeneous and flexible information infrastructure enables such diversity.

Another way in which the information infrastructure may be of value is by producing results that are perceived as more legitimate because they represent a community [32]. For instance, a group of radiologists that provides expertise consultation is part of a community that may assess the results produced by the members of the group using some kind of socially constructed recommender system. Such a system is socially constructed, as it reflects a congruence between the behavior of the members of the group of radiologists (legitimate entity) and the (assumedly) shared beliefs of the community; therefore legitimacy depends on a collective audience, although it is independent of particular observers [35]. In this sense, legitimacy is seen as a social judgment of acceptance, suitability and desirability [36]. A basic premise to this is that the individuals of the community have an identity to enable interaction and communication, and association with the information produced [32], as legitimacy is dependent on an individual’s history of events [35]. In fact, involvement of the community in building legitimacy for the radiology practice is essential so as to reinforce the growth and sustainability of the very community in the radiology social space, because legitimacy is an important factor for attracting resources from the external environment to maintain such growth and sustainability [36]. It is also important to empower patients in the relationship with radiology practice, either by providing information to support decision-making, such as choosing an expert for a second opinion, or by offering the possibility to evaluate the actions of a professional.

Finally, the information infrastructure may be of value by executing tasks that require exclusively human abilities, beyond the capacity of purely IT systems [32]. This is the case of interpreting medical images, a complex task that IT systems generally cannot perform alone. The task involves the process of image perception to identify abnormal patterns, followed by characterization and interpretation of those patterns [37], which depend heavily on empirical knowledge, memory, intuition, and diligence of the radiologist [38]. Despite this, computer-aided diagnosis (CAD) can be a helpful tool to support the radiologists in decision making, particularly in the process of identifying abnormal patterns. For example, studies have demonstrated improved diagnostic sensitivity with the use of CAD for assessing breast nodules, although with increased false-positive results [37]. This CAD could be useful in large-scale breast screening programs to pre-select imaging studies where possible breast nodules were detected, to distribute them among a taskforce of radiologists from groups of subspecialists who provide expertise consultation to the social radiology information infrastructure. The radiologists would use the CAD results as a “double-check” and in such a case, with increased false-positive results, this may help to reduce inter-observer variability among radiologists [37]. It is noteworthy that the final decision lies with the radiologists, providing additional value due to the synergistic effect of combining the radiologist’s skills and the IT system’s capability [39].

##  Towards Social Radiology as an Information Infrastructure

This article explores the concept of social radiology as an information infrastructure, showing that the persistent tension between the local and global demands for radiology practice is an impediment for the emergence of such an information infrastructure. Tension persists due to the inertia of locally installed bases in radiology departments, for which common teleradiology models are not truly capable of reorganizing as a global social space for radiology practice. Reconciliation between local and global will facilitate the sharing of medical imaging studies beyond local domains, allowing the spontaneous formation of temporary or permanent practice alliances of (groups of) health professionals and organizations in a flexible and dynamic manner. With this reconciliation, the conceptual boundaries between (local) PACS and (global) teleradiology will vanish, signaling the emergence of social radiology as an information infrastructure.

The challenge is how to induce a movement to build social radiology as an information infrastructure, considering that it involves addressing a variety of issues, which are beyond the local and global tension examined in this article, for example, the tension between social and technical demands for radiology practice that arises among members of users and design communities, governance and standardization bodies, and health care organizations. More specifically, this tension is present in the sociotechnical process of developing information infrastructure standards that increases irreversibility in the use of technologies (eg, DICOM) while being open to further change and supporting flexibility of use [40]. This sociotechnical tension is also present in the relationship between user and open source software communities with traditional companies of medical imaging software [41]. Individual versus community demands are also a source of tension [2], and must be addressed in the move to build social radiology as an information infrastructure as well as security questions concerning the establishment of trust among the sociotechnical entities comprising the information infrastructure.

In the face of all these issues, recent advances in information infrastructure research [1-7], particularly in information infrastructure design theories [42], provide a promising way towards solutions for building social radiology as an information infrastructure. The design theory for dynamic complexity in information infrastructure [1] is a normative design theory systematized from empirical descriptions of the evolution of information infrastructures that tackles dynamic complexity in the design for information infrastructures, defined as a sociotechnical system of information technology. According to the proposed theory, information infrastructures have evolutionary dynamics that are nonlinear, path dependent and influenced by unbounded user and designer learning, as well as by network effects. In addition, information infrastructures are regulated by emergent, distributed, episodic forms of control. Therefore, information infrastructure design theory is aligned with a new view of health systems (such as a social radiology information infrastructure) as complex adaptive systems [17,43]. However, more research is needed regarding the application of design theories aimed at building information infrastructures for health systems, especially, social radiology.

## Abbreviations

CAD: computer-aided diagnosis
CNPq: National Counsel of Technological and Scientific Development
DICOM: Digital Imaging and Communications in Medicine
ICT: information and communication technology
INES: National Institute of Science and Technology for Software Engineering
IT: information technology
JPEG: Joint Photographic Experts Group
MIME: multipurpose internet mail extensions
NM: nuclear medicine
OpenPGP: open pretty good privacy
PACS: picture archive and communication systems
RIS: radiology information systems
VPN: virtual private networks
WAN: wide area network
